# The social and cultural construction of psychiatric knowledge: an analysis of NICE guidelines on depression and ADHD

**DOI:** 10.1080/13648470.2012.747591

**Published:** 2013-03-18

**Authors:** Joanna Moncrieff, Sami Timimi

**Affiliations:** a Department of Mental Health Sciences, University College London, London, UK; b Lincolnshire Partnership NHS Foundation Trust, UK

**Keywords:** depression, ADHD, evidence based medicine, clinical guidelines, sociology of knowledge

## Abstract

The current paper presents an analysis of the NICE guidelines on depression and attention deficit hyperactivity disorder (ADHD) from the perspective of the philosophy of science, guided particularly by Foucault's notion of the symbiosis of knowledge and power. It examines how data that challenged the orthodox position on the validity and drug treatment of these conditions was managed in the process of guideline development. The depression guideline briefly considered the complexity and heterogeneity of depression, and numerous methodological problems with evaluating treatments, including antidepressants. However, the guideline recommendations made no reference to these issues and ignored evidence that questioned the analysis of antidepressant trials. The guideline on ADHD reviewed validity, but did not consider evidence from the critical literature, and overlooked inconsistencies in the data. The guideline identified that drug trials have shown no long-term benefit in ADHD, but still recommended treatment with stimulant drugs for children with severe symptoms and for all adults claiming consensus for this position. Both guidelines demonstrate how contradictory data are managed so as not to jeopardise the currently predominant view that ADHD and depression are valid and un-contentious medical conditions that should be treated with drugs. The subjective nature of guideline formation that is revealed illustrates Foucault's suggestion that the authority of medicine operates to promote a technological view of the nature of certain human problems, which in turn strengthens medical hegemony over these areas.

## Introduction

The National Institute for Health and Clinical Evidence (NICE) was set up by the government of the United Kingdom in 1999 with a remit to make evidence-based recommendations about particular medical conditions and their treatment. Since then, NICE guidelines have become internationally influential and, unless appropriately contested, are often viewed as representing the gold standard of medical practice.^1^

The NICE guideline on depression was first published in 2004 and updated in 2009 (National Institute for Health and Clinical Evidence 2004, 2009). It recommended that antidepressants should be prescribed in moderate to severe cases of depression, but that mild depression should be treated without medication, at least initially. In September 2008, the NICE guideline on Attention Deficit Hyperactivity Disorder (ADHD) was published (National Institute for Health and Clinical Evidence 2008a). The guideline concluded that both childhood and adulthood ADHD were ‘valid’ diagnoses and recommended that stimulant drugs should be the initial treatment for all adults with ADHD and children with the most severe symptoms.

The increasing popularity of clinical guidelines reflects political changes in western systems of healthcare delivery, and the changing nature of professional power (Harrison 1996). Organisations now frequently require healthcare to be delivered according to guideline recommendations, for example, restricting professional autonomy. Research, however, has highlighted the ‘divergence and conflict’ present within the process of guideline formation (Moreira 2005). In other areas, it has been shown how guidelines privilege certain forms of knowledge and research designs above others (Lambert 2006; Goldenberg 2006), and they often ignore the social and political influences on the formation and interpretation of evidence (Goldenberg 2006). Empirical studies of the process of formation of guidelines confirm that factors other than evidence influence the content and recommendations, including physician enthusiasm (Jenkings and Barber 2004), previous practices (Landesman 2006) and political acceptability (Moreira 2005).

Philosophers of science and knowledge have long questioned the idea of objective, value-free, scientific knowledge that is embodied in clinical guidelines. They have pointed out that knowledge is shaped by local and historical circumstances (Kuhn 1970; Pickering 1992; Latour 1987), and that it reflects the moral and political values of the society in which it is produced, as well as the interests of particular groups within that society (Kuhn 1970; Feyerabend 1975; Proctor 1991). Foucault's examination of the emergence of modern psychiatry indicates that psychiatric knowledge, in particular, represents moral and political values that have been ‘overlaid by the myths of positivism’ (Foucalt 1965, 276). Foucault used psychiatric knowledge to illustrate the association of knowledge and power. It is the ‘medical authority’ of the psychiatric profession, which ‘functions as power, well before it functions as knowledge’ (Foucault 2006, 3) that enables it to define what counts as knowledge in psychiatry. This knowledge in turn ‘founds the rights of this power’ (Foucault 2006, 346).

The current paper presents a systematic examination of the process of guideline formation, using the guideline documents themselves, and some direct process data (see below). The analysis focuses on how NICE managed information that contradicted or challenged the currently predominant view that depression and ADHD are valid concepts and that the disorders they represent can be successfully treated with drugs. The analysis illustrates the way that psychiatric knowledge is shaped more by human interests and power dynamics than objective data.

## Methods

From its inception, NICE offered that any interested groups or organisations could become registered ‘stakeholders’. Stakeholder groups are invited to comment on a draft of guidelines relevant to their interests prepared by a Guidelines Development Group, made up of invited experts and service user representatives. The Guideline Development Group considers these comments during the process of formulating the final draft of the guidelines and makes a detailed response to the stakeholders about the comments submitted.

The authors of the current paper were involved in commenting on drafts of the depression and ADHD guidelines on behalf of the Critical Psychiatry Network, a registered stakeholder.^2^ One of the authors (ST) also participated in a ‘conference of experts’, organised by the NICE Guideline Development Group to inform the development of the ADHD guideline. The Full guideline itself, as well as the draft version, can be also viewed as process data, since they describe what evidence was selected for consideration by the Guideline Development Group, what evidence was omitted, and how this evidence was managed in relation to the final recommendations. The current paper is based on an in-depth analysis of the guideline documents, the NICE response to the Critical Psychiatry Network's contributions (hereafter referred to as the stakeholder contributions), and the experience of the second author during and after the guideline conference.

## Management of conceptual and evidential issues in the Depression Guideline

### Discussion of conceptual issues

In sections dealing with the concept of depression, the NICE guideline acknowledges that there are some difficulties in defining the term, and in categorising severity, but these difficulties and their implications are not examined in depth and do not influence subsequent discussions or recommendations. The presence of some discussion, however cursory, allows the guideline to claim that the question of validity has been covered, without jeopardising the subsequent analysis and conclusions.

The introduction of the guideline states that ‘Distinguishing the mood changes between major depression and those occurring “normally” remains problematic’ (National Institute of Health and Clinical Excellence 2004, 14) and later that ‘the initial presentation and form of depressive illness varies considerably’ (National Institute of Health and Clinical Excellence 2004, 15), pointing out that symptoms include opposite physiological states such as increased and reduced sleep and increased and reduced appetite. When discussing diagnosis, the guideline notes that although the introduction of diagnostic criteria has improved the reliability of diagnosis, ‘there has been no parallel improvement in the validity of diagnosis’ (19). Later in this section it is suggested that

there is increasing concern that ‘depression’ may be too heterogeneous in biological, psychological and social terms to enable clarity on which specific interventions will be effective – for which problem, for which person, and in which context. (19)

Despite the uncertainty expressed in these quotations, the guideline did not consider any critical literature on the validity of the concept of depression, despite references being provided in the stakeholder submissions (Pilgrim and Bentall 1999; Dowrick 2004).

The Guideline recommendations categorise depression into ‘mild’, ‘moderate’ and ‘severe,’ based on the tenth edition of the International Classification of Disease (ICD-10) (World Health Organisation 1990) and treatment recommendations are based on this subdivision. ICD-10 defines categories of severity according to how many concurrent symptoms are present. The guideline goes on to suggest that

it is doubtful whether the severity of a depressive illness can realistically be captured in a single symptom count, although there is some evidence for this. (Faravelli et al. 1996, 19)

The paper it cites as evidence in this sentence shows that the number of symptoms does not correlate with the severity of a depressive disorder when severity is measured by non-symptom count approaches such as measures of global clinical state and functioning (Faravelli et al. 1996).

### Resolving conflicting evidence: drug treatment of depression

In a similar approach, NICE discusses the methodological problems of antidepressant trials in the Full Guideline, discussing at some length publication bias, infringement of the double blind, and uncertainty about the generalisability of trial subjects. The guideline then moves on without probing too far into the implications of these problems. These, and other issues relating to methodological problems in trials that were raised in the stakeholder submission, were either not commented on or given a brief and superficial response in the guideline development group's reply to comments submitted for the first version of the Guideline produced in 2004. No response was ever made to comments made in the stakeholder submission for the revised Depression guideline published in 2009. Hence, NICE did not explain why studies that showed that early detection of depression did not improve outcome, and well-known negative antidepressant studies and meta-analyses were not cited, and it did not engage with comments made about the inconsistency of its claims about severity and antidepressant response. These failures to include significant material allowed NICE to present clear and unambiguous recommendations on drug treatment.

The most important point made in the stakeholder submission on the draft guidelines concerned the presentation of data from the meta-analysis of placebo controlled trials of SSRI antidepressants conducted by NICE, in particular the use of categorical data. In this analysis, reduction in depression rating scale scores over the course of treatment were slightly greater in people taking SSRIs, but the guideline authors considered that, although statistically significant, the differences were so small that they were ‘unlikely to be of clinical significance’ (National Institute of Health and Clinical Excellence 2004, 169). The guideline went on to present data on response rates, which were interpreted as showing statistically and clinically significant differences. However, these response rates were produced by categorising the same data on depression rating scale scores that, when examined as a continuous variable, had produced the findings that were considered to be of no clinical significance. In the introductory section of the Full Guideline it is acknowledged that categorising data can create an ‘artificial boundary’ with patients just over the cut-off score often being clinically indistinguishable from those just under the cut-off’ (National Institute of Health and Clinical Excellence 2004, 38). Despite this statement, the major recommendations that relate to the use of antidepressants (SSRIs) in the NICE guideline were based on the analysis of response rates. In the stakeholder submission, a detailed statistical example was presented, using hypothetical data, to demonstrate how differences in mean scores can be inflated by the categorisation of data using an arbitrary cut off score (Kirsch and Moncrieff 2007). In its response to the stakeholder's comments, the guideline development group, which made some sort of response to every other comment, left this box blank. No changes were made to the use and interpretation of categorical data in the final guideline.

In a similar manner, whilst NICE admits that patients with mild depression should be treated without antidepressants, it recommends that those with moderate or severe depression should receive medication. The Full guideline, however, admitted that there is little evidence with which to relate antidepressant effects to severity levels (National Institute of Health and Clinical Excellence 2004, 163), and NICE's own meta-analysis, found that differences between antidepressants and placebo were greatest in the middle category of severity, not the highest. The recommendations of the Guideline, therefore, do not reflect the evidence presented, and other contradictory evidence highlighted in the Stakeholder submission was not cited.

## Management of conceptual and evidential issues in the guideline for attention deficit hyperactivity disorder

### Conflicts of interest

The Guideline Development Group that produced the NICE guidelines on ADHD was made up of academic psychiatrists and others known to be strident advocates of the concept of ADHD, including the foremost exponent of the diagnosis of ‘adult ADHD’ in the United Kingdom. Much of the research cited in the guideline was conducted by a small number of researchers including members of the Guideline Development Group and their associates and several members of the group declared financial interests involving pharmaceutical companies.

### Representing dissent

During the early stages of guideline development, what was then referred to as a ‘conference of experts’ was set, at which one of the authors of this paper, was asked to participate (ST). ST stated, prior to accepting the invitation that he feared that he would be used as the ‘token’ critic to enable NICE to state that they had represented a cross-section of professional opinion, and was reassured that this would not be the case. During the meeting, which was supposed to focus on the validity of the diagnosis, ST presented a detailed argument, that the neurodevelopmental model of ADHD could not be supported by existing evidence. The questions from the guideline development group following this presentation, did not focus on any of the content of the presentation, but instead was dominated by repetitive questioning about whether or not ST had ever prescribed stimulants. Despite ST providing only qualified assent to this question, in that he pointed out that he had not initiated a prescription of stimulants for a new patient for many years, the Full Guideline described the meeting as a ‘consensus conference’ and the report emphasises repeatedly the agreement and ‘unanimity’ between participants, particularly with regard to the Guideline recommendation for the drug treatment of children with severe symptoms (National Institute for Health and Clinical Excellence 2008a, 119). As ST was the sole practising clinician invited to the conference who had a critical view of the concept of ADHD and its treatment, the admission that he had prescribed stimulants appears to be the basis for this claim of consensus. The conference report plays down the level of disagreement between him and other attendees, and thus misrepresents his views on stimulant treatment, claiming consensus and unanimity where there was none.

### Dealing with controversy and inconclusive evidence: validity of ADHD

As for depression, the recommendations section of the Full Guideline and the Quick Reference Guide on ADHD make no reference to there being any concern or controversy over the concept of ADHD. However, the text of the Full Guideline discusses the issue of validity at length. Although it concludes that ADHD is an easily identifiable, discrete disorder that responds to specific treatments, it acknowledges that:

The use of the diagnosis of ADHD has been the subject of considerable controversy and debate and the diagnosis itself has varied across time and place as diagnostic systems have evolved. (National Institute for Health and Clinical Excellence 2008a, 106)

Despite this caveat, the Full Guideline includes no references drawn from authors who are critical of the concept of ADHD, and issues such as the cross-cultural validity, gender disparity, and social class distribution of ADHD were largely ignored. Moreover, there is no agreement about what level of symptoms could be expected as a baseline in children or adults without the disorder. Despite this, even by its own standards, the guideline provides little evidence that ADHD satisfies the validity criteria specified, and, like the depression guideline, it glosses over apparent problems and often fails to mention contradictory data.

The guideline specifies the following validity criteria: that the symptoms of ADHD (as specified in diagnostic systems such as the ICD 10, and the American Diagnostic and Statistical Manual version IV) should cluster together and be distinguishable from normal variation and other psychiatric conditions; that symptoms are associated with significant clinical or social impairments; that symptoms are associated with a characteristic temporal pattern or outcome and that there is ‘consistent’ evidence of genetic, environmental or neurobiological risk factors.

The guideline development group reviewed studies using factor analysis and latent class analysis of symptoms of behavioural disorders in children. The majority of studies using factor analysis found that ADHD symptoms ‘represent separate but correlated factors from oppositional and conduct problems’ (National Institute for Health and Clinical Excellence 2008a, 102). However, the Guideline does not mention that two large studies using Latent Class Analysis failed to find a clear distinction (Sondeijker et al. 2005; van Lier et al. 2003). Other literature, not considered in the Guideline, suggests that it is also difficult to distinguish ADHD in children and adolescents from depression, obsessive-compulsive disorder and anxiety (Jensen, Martin, and Cantwell 1997) and that about two thirds of children diagnosed with ADHD have a co-morbid diagnosis (Dulcan and Benson 1997).

The Guideline concludes that it is also difficult to distinguish ADHD from the normal spectrum of behaviour, since there is much overlap between people with and without the diagnosis in terms of symptoms or behaviours: ‘Most analytic approaches are unable to make a clear distinction between the diagnosis of ADHD and the continuous distribution of ADHD symptoms in the general population’ (National Institute for Health and Clinical Excellence 2008a, 104).

Some of the extensive literature on the association between ADHD and a variety of impairments including academic, familial, social and behavioural impairments are presented in the Guideline, which concludes that a ‘high level of ADHD symptoms is associated with impairment in multiple domains’ (National Institute for Health and Clinical Excellence 2008a, 107). However, it failed to cite any evidence that *symptoms*, rather than the diagnosis, are associated with impairments. Since the diagnostic criteria for ADHD require the presence of ‘significant levels of impairment’ (National Institute for Health and Clinical Excellence 2008a, 96), studies that report impairments in people diagnosed with ADHD are simply tautological. The evidence reviewed also demonstrates the difficulties of distinguishing the role of ADHD and co-morbid conduct disorder. Although some studies cited suggest that a diagnosis of ADHD is independently associated with impairments (Frick et al. 1991; Mannuzza 2004), many more studies, some cited and some not, suggest that co-morbidity with conduct disorder, together with adverse environmental conditions (such as socio-economic disadvantage, maternal depression, and marital discord), rather than ADHD severity, are associated with the most adverse outcomes (Barkley et al. 2004; Fergusson, Horwood, and Ridder 2007; Biederman et al. 1996; Lee and Hinshaw 2004).

The Guideline emphasises the stability of symptoms throughout childhood as evidence that the disorder has a characteristic pattern and outcome. However, three of the four studies cited show only ‘moderate’ stability, which is represented, for example, by a correlation of only 0.5 between symptoms at different ages (Price et al. 2005).

As far as aetiological factors are concerned, NICE follows prominent academics in the world of child psychiatry, by implicitly referring to ADHD as a ‘neurodevelopmental disorder’ (i.e. a disorder of the development of the nervous system), suggesting for example, that ‘… ADHD may be viewed as one component of a general propensity to neurodevelopmental problems’ (National Institute for Health and Clinical Excellence 2008a, 103). Genetic and neuroimaging studies are cited to support this position, but other statements appear to contradict this view. In the introduction to the Full Guideline, for example, NICE acknowledges that:

‘Nevertheless, the disorder remains one that is defined at a behavioural level, and its presence does not imply a neurological disease’ (National Institute for Health and Clinical Excellence 2008a, 15) and ‘the diagnosis of ADHD does not imply a medical or neurological cause’ (National Institute for Health and Clinical Excellence 2008a, 28).

In any case, the research cited only provides evidence of a weak genetic link, and no data are presented that could exclude confounding factors, or confirm the specificity of the associations described. The review of genetic studies concludes that: ‘As with all other types of risk factors associated with ADHD, the individual genetic variants associated with the disorder are neither sufficient nor necessary to cause it, but contribute a small increase to the overall risk for ADHD’ (111). The Guideline Development Group did not consider criticisms of the genetic studies, however, including possible confounding with co-morbid conditions such as learning disability and the fallacy of the ‘Equal Environment Assumption’ in twin studies (the assumption that identical and non identical twins have equivalent environmental influences) (Joseph 2003).

NICE notes the lack of consistency found in neuroimaging studies, which suggests that numerous brain regions may be implicated, with little replication of regions between studies. The guidelines also notes that ‘it was not possible to include or exclude the role of medication in the observed changes to brain volume and structure’ (National Institute for Health and Clinical Excellence 2008a, 110), but there is no comment on other possible confounders such as intelligence, or on the overlap with findings in other psychiatric conditions.

The guideline also concludes that there is a positive association with a large number of familial and environmental adversity indicators, including prenatal maternal stress, early deprivation, maltreatment, sexual abuse, large family size, paternal criminality, maternal mental disorder, foster care placement, and some dietary components. Again, however, no evidence is offered that any of these factors are specific to ADHD, as opposed to say conduct disorder, and the large number of associated factors suggests that no specific cause has actually been identified. If everything causes ADHD, then in fact we know nothing about what causes it.

### Managing a lack of evidence: adult ADHD

Since adult ADHD is a recent construct, NICE might be expected to consider the issue of validity particularly closely. Instead, the Guideline accepts the diagnosis with little question. First, the guideline cites one meta-analysis suggesting that 15% of children with ADHD continued to fulfil diagnostic criteria at age 25 (Faraone, Biederman, and Mick 2006). It continues:

The profile of symptoms may alter with a relative persistence of inattentive symptoms compared with hyperactive-impulsive symptoms; however the evidence base for this conclusion is poor and based on the analysis of developmentally inappropriate measures of hyperactivity-impulsivity in adults. The GDG (guideline development group) concluded that there is currently insufficient evidence to warrant a different diagnostic concept in childhood and in adulthood. (National Institute for Health and Clinical Excellence 2008a, 108)

Somewhat unusually, this, the main section supporting the validity of adult ADHD, does so on the basis of a *lack* of evidence that the concept of adult ADHD is different to childhood ADHD, offering no positive evidence of direct links between the two conditions. There is also little acknowledgement that ADHD has its origins as a childhood disorder and that concepts such as impulsivity and hyperactivity were originally defined as developmental problems, their applicability to adults remaining uncertain.

The only criterion of validity that was examined in relation to adult ADHD was whether it can be distinguished from other common adult psychiatric disorders. The Guideline notes the overlap between symptoms suggested to be characteristic of adult ADHD and symptoms of various personality disorders (National Institute for Health and Clinical Excellence 2008a, 102, and 129–30) and cites the United States National Comorbidity study that found substantially increased rates of mood disorders, anxiety disorders, substance use and impulse control disorders in people diagnosed with adult ADHD (Kessler et al. 2006). No evidence is presented that suggests that adult ADHD can be distinguished from any of these overlapping conditions.

### Selecting the evidence: the use of stimulants

In common with the depression guideline, and with other systematic reviews of ADHD medication treatment (Schachter et al. 2001; King et al. 2006), the NICE review notes the inadequate reporting of drug trial methodology, possible publication bias, limited reliability of results, inadequate data regarding adverse events, and lack of evidence of long-term benefit, concluding that the evidence does not support using medication as a first line treatment for mild or moderate ADHD in children. Yet, on the basis of the same trials, as well as citing expert opinion, NICE concludes that medication should be used as a first line treatment in ‘severe’ ADHD.

The guideline states that

Even the most ardent supporters of non-pharmacological interventions in ADHD recognised the importance of pharmacological treatment in the most severe cases. (National Institute for Health and Clinical Excellence 2008a, 121)

No reference is provided for this statement, which appears therefore to be based on the guideline development group's representation of the ‘consensus conference’ detailed above.

The second reason given in support of using medication as a first line treatment in severe ADHD referred to a paper that reports a re-analysis of data from the largest trial comparing medication and behavioural treatments (the Multimodal Treatment Study of children with ADHD, or MTA study). This paper concluded that a subgroup of children with more severe ADHD symptoms showed a larger decrease in symptoms with medication than with behaviour therapy (Santosh et al. 2005). The MTA study has been criticised on a number of grounds, including the fact that most ratings were not conducted blind to treatment group and that a few positive measures were selectively highlighted, with more numerous negative findings being ignored (Breggin 2001). In addition, data used for the severity analysis was gathered at the 14-month point of the study and data obtained after three years’ follow up did not reveal beneficial long-term effects of medication over behaviour therapy, even in those with more severe symptoms at the start (Jensen et al. 2007). In reply to the stakeholder submission, NICE chose not to comment on the paucity of evidence for stimulants being more effective with ‘severe’ ADHD beyond the following non-specific statement: ‘[We] do see that psycho stimulants have a place which we specify very carefully and based upon the evidence’ (National Institute for Health and Clinical Excellence 2008b).

The recommendation of the NICE guideline to use medication as a first line treatment for adult ADHD is based on three studies with study duration of 21–45 days (Biederman et al. 2006; Spencer et al. 2005; Kooij 2004). Two of these studies were conducted by a group of researchers based at Harvard, some of whom were recently revealed to have received million of dollars of personal income from pharmaceutical companies, which they had not declared (Harris and Carey 2008). A recent meta-analysis found that there was no difference between stimulants and placebo in parallel group randomised trials in adults with ADHD, and that studies conducted by the Harvard group reported considerably larger effect sizes than other studies (Koesters et al. 2008).

## Discussion

The analysis of two NICE guidelines presented here shows how NICE managed challenges to the validity of the concepts of depression and ADHD, and claims for the efficacy of drug treatment, by acknowledging, but never seriously engaging with, opposing arguments and evidence. This strategy enabled NICE to employ the rhetoric of consultation, while effectively marginalising dissenting views, in order to produce an impression of consensus.

NICE's recommendations on the treatment of depression and ADHD make no reference to any problems with the concepts concerned, implying their validity is universally accepted and unproblematic. The depression Guideline, for example, recommends screening certain populations for the presence of depression, which implies a confidence in the diagnosis of depression that is belied by statements in other parts of the Guideline itself. Other than acknowledging that drug treatment of milder disorders is unsupported, recommendations on drug treatment in both Guidelines also failed to reflect the methodological difficulties that were described in other parts of the Guidelines, and the consequent uncertainty of the evidence. Consensus for drug treatment of severe ADHD was claimed without justification and comments that challenged the interpretation of evidence on antidepressants in the depression guideline were ignored.

In both depression and childhood ADHD, the guidelines do not recommend drug treatment as a first line in mild cases, but only as a first line treatment in severe, or moderate to severe, cases. However, although the idea that drug treatment is likely to be more effective in more severe conditions has been assumed for many years, little evidence is cited in either guideline to support this supposition. Since the criteria for assessing severity are only loosely defined, have no distinguishing features, and are not used clinically, it is likely that the label ‘severe’ will simply be applied to cases in which drug treatment is the desired outcome of the diagnoser or service user. The fact that by 2010, prescriptions for antidepressants in England had risen by 48% to 43 million since the publication of the NICE guideline in 2004, and that stimulant prescriptions have continued to rise since 2008, supports this notion (Information Centre for Health and Social care 2009).

The recommendations concerning adult ADHD are perhaps the most remarkable. A sea change in current psychiatric practice is recommended, with encouragement for the use of a controlled drug with a potentially large adult population and where the possible personal and public health implications of this remain unexplored. The case for the validity of the disorder was hardly considered, and the evidence cited in support of drug treatment was slim and contentious.

The current analysis focuses on the content and development of the NICE guidelines, and has not looked in detail at underlying motivations and interests. The conclusions of both guidelines support the primacy of a technological, medically-based approach to understanding and treating psychiatric disorders, and as others have described, a complex array of interests are served by the medicalisation of human difficulties (Conrad 2009). The medical profession and the pharmaceutical industry obviously benefit, but patients and parents have also been instrumental in the medicalisation of ADHD in particular (Conrad and Potter 2000). Some of the authors of both guidelines considered here have declared financial conflicts of interest involving various drug companies, in common with authors of other clinical guidelines (De Vries and Lemmens 2006; Healy 2006). Although recommendations against the use of drugs in mild disorders may appear to represent a setback to drug sales, by deflecting criticism of the overuse of drugs, they may paradoxically increase the legitimacy of the use of drugs for behavioural problems in general. The recommendation of stimulant use for adult ADHD is a significant boon to the industry, which has recently identified adult ADHD as a fertile area for increased sales (Lead Discovery 2004).

The current analysis of the formation of psychiatric guidelines illustrates the symbiosis between power and knowledge highlighted by Foucault (2006). NICE guidelines are not unbiased, value-free accounts that arise unaided from the data. They represent a particular position rooted in a technological understanding of the problems concerned, a position that guided and shaped the selection and interpretation of evidence and excluded or ignored evidence that was contradictory. When the Guidelines are published, their influence and authority further entrench the biomedical framework, legitimising the treatment of behavioural and emotional disorders with psychotropic drugs, and eclipsing other accounts of these problems (Moncrieff 2008). Viewing guidelines in this light raises questions about the ethics of this process of guideline formation, which can result in the increasing use of drug treatments of unproven value in adults and children with a variety of problems.

Revealing the mechanics of guideline construction, and the difficult task of moulding divergent views and flawed and inconsistent evidence into an authoritative justification of practice, helps to underline the contested nature of psychiatric ‘knowledge’. Further investigation could help to illuminate the diverse interests that are served by the medicalising perspective that the guidelines endorse.
